# Foodborne disease outbreak in a resource-limited setting: a tale of missed opportunities and implications for response

**DOI:** 10.11604/pamj.2016.23.69.7660

**Published:** 2016-03-09

**Authors:** Donne Kofi Ameme, Marijanatu Abdulai, Eric Yirenkyi Adjei, Edwin Andrews Afari, Kofi Mensah Nyarko, Dwamena Asante, Gideon Kye-Duodu, Mona Abbas, Samuel Sackey, Fred Wurapa

**Affiliations:** 1 Ghana Field Epidemiology and Laboratory Training Programme (GFELTP), School of Public Health, University of Ghana, Accra, Ghana; 2 Upper West Akim District Health Directorate, Adeiso, Ghana; 3 School of Public Health, University of Health and Allied Sciences, Hohoe, Ghana; 4 Noguchi Memorial Institute for Medical Research, Legon, Accra, Ghana

**Keywords:** Foodborne disease, outbreak, investigation, retrospective cohort

## Abstract

**Introduction:**

Foodborne diseases (FBD) have emerged as a major public health problem worldwide. Though the global burden of FBD is currently unknown, foodborne diarrhoeal diseases kill 1.9 million children globally every year. On 25th September 2014, health authorities in Eastern Region of Ghana were alerted of a suspected FBD outbreak involving patrons of a community food joint. We investigated to determine the magnitude, source and implement control and preventive measures.

**Methods:**

A retrospective cohort study was conducted. We reviewed medical records for data on demographics and clinical features. A suspected foodborne disease was any person in the affected community with abdominal pain, vomiting and or diarrhea between 25^th^ and 30^th^ September 2014 and had eaten from the food joint. We conducted active case search, descriptive data analysis and calculated food specific attack rate ratios (ARR) and their corresponding 95% confidence intervals.

**Results:**

Of 43 case-patients, 44.2% (19/43) were males; median age was 19 years (interquartile range: 17-24 years). Overall attack rate was 43.4% (43/99) with no fatality. Case counts rose sharply for four hours to a peak and fell to baseline levels after 12 hours. Compared to those who ate other food items, patrons who ate “waakye” and “shitor” were more likely to develop foodborne disease [ARR = 4.1 (95% CI = 1.09-15.63)]. Food samples and specimens from case-patients were unavailable for testing. Laboratory diagnostic capacity was also weak.

**Conclusion:**

A point source FBD outbreak linked to probable contaminated “waakye” and or “shitor” occurred. Missed opportunities for definitive diagnosis highlighted the need for strengthening local response capacity.

## Introduction

Foodborne diseases (FBD) encompass a wide array of illnesses caused by ingestion of foodstuffs contaminated with microorganisms or chemicals [1]. Contamination usually occurs during food preparation or serving but may occur at any point from farm to fork. Though preventable [2], FBD remain an important cause of morbidity and mortality, posing a major public health challenge globally. They contribute to marked economic loss, reduction in quality of life and productivity [3]. Currently, there are no reliable estimates for the burden of FBD globally. However, diarrhoeal diseases alone-which form a sizeable proportion of FBD-kill 1.9 million children globally every year [4]. The presentation of FBD is varied with gastrointestinal illness being the commonest; though extra gastrointestinal presentation has been recorded [5]. Contamination usually occurs during food preparation or serving but may occur at any point from farm to fork. Food handlers have been implicated in many FBD [6]. Though FBD may be severe enough to warrant hospitalization, they could be mild [7] or self-limiting [8] typically lasting for few days. The true incidence of FBD is usually under estimated as minor and sporadic outbreaks go unreported [9]. Misdiagnosis, improper sample collection and laboratory diagnosis [10], worsened by lack of seeking medical attention by affected persons [11], also contribute to the seemingly low incidence of FBD. Laboratory diagnosis is also complicated by lack of routine collection and testing of patients’ stool specimens for specific pathogens and their enterotoxins [12, 13]. In Ghana, these factors are aggravated by the non existence of an established surveillance system FBD. FBD outbreaks are subsumed in other public health events and therefore seldom reported. Even when reported, capacity of the local health officials to perform the initial critical steps in the response path is usually lacking. We report a FBD outbreak in a community in the Eastern Region of Ghana highlighting the missed opportunities for conclusive diagnosis and suggested remedial actions. On the 25^th^September 2014, at 19:00 hours Greenwich Mean Time, the Eastern Regional Disease Surveillance Officer was alerted by the Upper West Akim District Disease Control Officer of a suspected FBD outbreak in one of the communities in the district. A group of residents mostly students from a local community in Adeiso, in the Upper West Akim District of the Eastern Region of Ghana presented to the district health centre with complaints of sudden onset of abdominal pain, nausea, vomiting and diarrhoea. They had all eaten from a particular food vendor in the community on that particular day. The affected individuals had not eaten another common meal that day. The local health officials therefore suspected a FBD disease outbreak. The Eastern Regional Health Directorate was notified and a team constituted to commence investigations into the outbreak to determine the extent of the outbreak, identify the source and aetiologic agent and institute control and preventive measures.

## Methods

We conducted the outbreak investigation from the 27^th^ to the 30^th^ of September 2014 at Adeiso, the district capital of the newly created Upper West Akim District of the Eastern Region. Adeiso is about 55km from Accra, the capital of Ghana. Upper West Akim District has a population of 100,245. It is divided into seven sub-districts, which are served by 31 health facilities, including four health centres, five private clinics, one maternity home and 21 functional Community-based Health Planning and Services (CHPS) centres. The district has no hospital andbasic laboratory services are available only at the health centres. There are several food joints, which are patronized by the residents. The district's main water source is pipe borne water. The district, at the time of this outbreak investigation was among the many districts grappling with a nationwide cholera outbreak. Over 57 cases of cholera had been reported between July and September 2014 with no deaths. Surveillance activities in the district had therefore been enhanced.

### Data collection

We reviewed inpatient and outpatient health records at the District Health Centre and District Health Directorate. We abstracted data on age, sex, date of onset of illness, date of presentation at health facility, signs and symptoms and outcomes. We defined a suspected case of foodborne disease as any person in Adeiso with abdominal pain, vomiting and or diarrhea between 25^th^ and 30th September 2014 and had eaten food from the food joint. Using the case definition, we conducted active case search in the community to identify unreported cases and updated the line list with the variables of the identified case-patients. We interviewed the community members using a questionnaire to collect data on their source of food and water. We interviewed patrons of the implicated food joint to collect data on their demographic characteristics, specific food items consumed and presence or absence of symptoms. We also held a durbar at one of the schools which students were mostly affected, to educate the staff and students on the causes and prevention of foodborne diseases. We educated the community members on causes and prevention of FBD. We conducted an environmental assessment of the community to determine the sources of food and drinking water as well as sites of refuse disposal. We recorded the geographic coordinates of the location of all the case-patients using GPS Essentials software downloaded onto a portable mobile device. We visited the food vendor, inspected her premises for conditions under which food was being prepared and sold. The entire food production process from purchase of ingredients to food preparation was thoroughly reviewed. The food handlers were examined for skin lesions on their body, hands and forearm. We interviewed the food handlers, educated them on food hygiene and took their stool and nasopharyngeal samples for laboratory investigations. All samples were transported to the laboratory within six hours via triple packaging systems and transport media. We also visited and interviewed the index case. There was no food specimen available for examination when the team arrived on the field. Stool and vomitus samples were also not available for laboratory investigation.

### Descriptive epidemiology

We conducted descriptive and inferential statistical analysis. Univariate analysis was done and categorical variables were expressed as frequencies and relative frequencies. Continuous variables were expressed as median and interquartile range (IQR). We described the data in terms of time, place and person. We calculated the overall, age and sex specific attack rates, drew a spot map and an epidemic curve to show the distribution of the case-patients in the community by time of onset of illness. Using the time of food consumption and illness onset, we calculated the incubation period of the disease.

### Analytical epidemiology

Descriptive statistics showed that all the case-patients who presented at the Adeiso Health Centre and those found in the active case search had eaten from a particular food joint earlier in the day. Based on this, we suspected that the illness was associated with eating from the food vendor. We hypothesized that a particular food item from the food vendor was the likely vehicle of transmission of the outbreak. To test this hypothesis we conducted a retrospective cohort study among residents of the community who had eaten from the food vendor on the 25th September 2014. All the patrons of the food joint on that particular day who were available were included in the cohort study. They were interviewed on the particular food items eaten, time of purchase, time of eating and time of onset of illness using a structured questionnaire. The menu for the day was provided by the food vendor. We conducted bivariate analysis to show association between consumption of each particular food item and development of illness. We calculated the Attack Rate Ratios (ARR) and their corresponding 95% confidence intervals (CI) to show significant association of illness with different exposures (food items consumed). Data entry, cleaning and analysis was done using Epi-info version 7.

### Ethical considerations

All respondents provided voluntary informed consent. Permission was sought from the Eastern Regional Health Directorate, the Upper West Akim District Health Management Team, as well as the traditional and political leaders of the community and approval obtained before the study. This outbreak investigation was deemed a response to a public health emergency by the Ghana Health Service and hence did not receive formal review by Ethical Review Committees. Confidentiality was observed throughout the investigation. The report of the investigation has been communicated to the regional and district authorities.

## Results

### Distribution of cases by person

**Figure 1:**
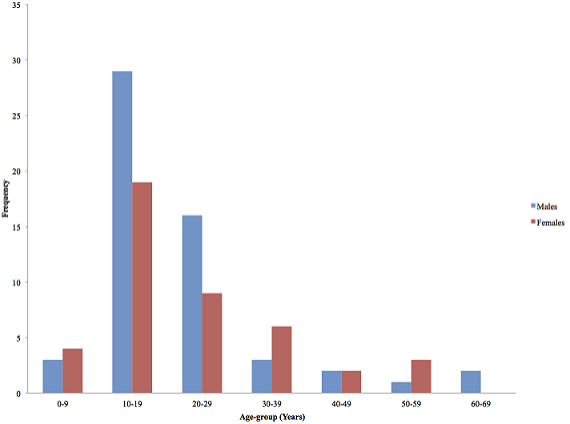
Age and sex distribution of foodborne disease case-patients in Adeiso, September 2014

**Figure 2:**
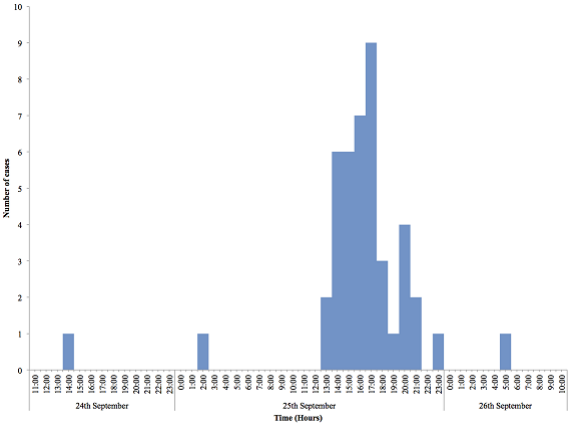
Foodborne disease by time of onset in Adeiso, 24th -26th September, 2014

**Figure 3:**
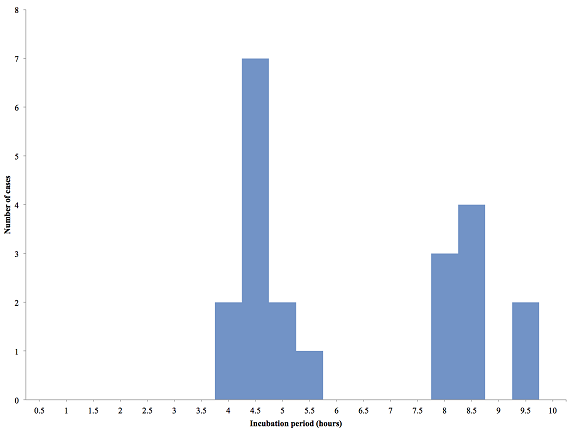
Incubation period of foodborne disease in Adeiso, September 2014

**Figure 4:**
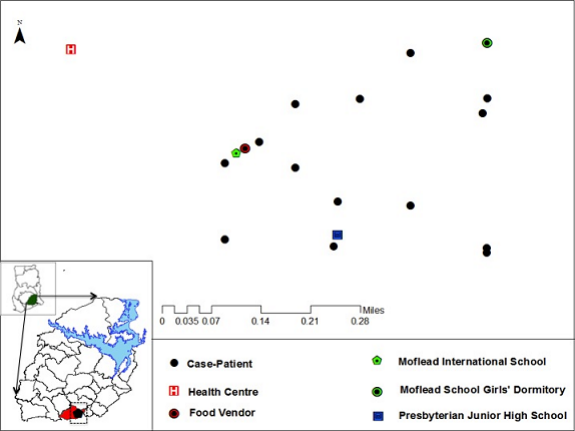
Geographical distribution of cases of foodborne disease in Adeiso, Upper West Akim District, September 2014

A total of 43 case-patients were identified of whom 44.2% (19/43) were males. The overall attack rate was 43.4% (43/99) with no fatality. Sex specific attack rates were 55.8% (24/43) and 33.9% (19/56) for females and males respectively. The median age of the case-patients was 19 years (IQR: 17-24 years). The most affected age group was 10-19 followed by 20-29 (Figure 1). More males were affected in the most affected age groups (10-19) and (20-19). The index case was a 34 year old male Senior High School teacher in the affected community who presented to the District Health Centre on the 25^th^ of September 2014 with abdominal pain, vomiting and watery diarrhea approximately 3-4 hours after eating food purchased from the popular food joint. He had not consumed any other meal that day. There was no fever. He was detained, managed and discharged the following morning. He was stable on the 29^th^ September 2014; vomiting and diarrhea had ceased but he complained of some residual abdominal pain for which he was seeking care at a private clinic. The epidemic curve of the outbreak shows a point source. One case occurred on the 24^th^ September 2014 at 14:00 hours followed by a second on the 25^th^September 2014 at 02:00 hours. The number of cases rose sharply to a peak at 17:00 hours on the 25^th^September and declined with the last case recorded on Friday 26^th^ September at 05:00 hours (Figure 2). The incubation period of the outbreak ranged from 4 hours to 9.5 hours (Figure 3). The median incubation period was 5 hours. Patrons who consumed food in the early hours of the day had relatively longer incubation period of 8 hours and beyond compared to those who ate late from the food joint. Majority 69.77% (30/43) of the case-patients were clustered around the vicinity of the food joint (Figure 4). Some households had more than one case patient.

### Analytical epidemiology

Compared to those who ate other food items, patrons who ate “waakye” and or “shitor” were 4.1 times more likely to develop foodborne disease at statistically significant levels (95% CI = 1.09-15.63). There was no difference between the risk of developing the foodborne disease among those who ate “gari”, salad and fish and those who did not eat these food items. Eating “ampesi” [ARR = 0.6 (95% CI = 0.27-1.55)], beef [ARR = 0.6 (95% CI = 0.11-3.50)], and “nkontomire” [ARR = 0.7 (95% CI = 0.33-1.40)] were found to be protective. These relationships were however not statistically significant (Table 1).

**Table 1:** Food specific attack rates of foodborne disease in Adeiso, September 2014

Number of persons who ate a specific food item				Number of persons who did not eat a specific food item				
Food item	Ill	Total	Attack Rate%	Ill	Total	Attack Rate%	Attack Rate Ratio	95% CI
“Waakye”	34	74	45.95	2	18	11.11	***4.1***	***1.09-15.63***
Rice	13	31	41.94	23	61	37.70	1.1	0.00-1.88
“Ampesi”	4	15	26.67	32	77	41.56	0.6	0.27-1.55
“Gari”	6	15	40.00	30	77	38.96	1.0	0.52-2.03
Spaghetti	21	35	60.00	15	57	26.32	***2.3***	***1.37-3.80***
“Wele”	1	1	100.00	35	98	35.71	***2.8***	***2.15-3.65***
Salad	7	18	38.89	29	74	39.19	1.0	0.52-1.89
Fish	9	23	39.13	27	69	39.13	1.0	0.56-1.80
Beef	1	4	25.00	35	88	39.77	0.6	0.11-3.50
Egg	14	29	48.28	22	63	34.92	1.4	0.83-2.29
“Nkontomire”	6	21	28.57	30	71	42.25	0.7	0.33-1.40
Tomato Gravy	30	66	45.45	6	26	23.08	2.0	0.93-4.17
“Shitor”	34	74	45.95	2	18	11.11	***4.1***	***1.09-15.63***
Sachet Water	20	43	46.51	16	49	32.65	1.4	0.85-2.38
“Waakye” and “Shitor”	34	74	45.95	2	18	11.11	***4.4***	***1.09-15.63***

“Waakye”: Local delicacy of boiled rice and beans

“Ampesi”: Boiled yam or plantain

“Gari”: Roasted grated cassava eaten raw, or with other food items

“Wele”: Fermented cow hide cooked and usually served in sauce

“Nkontomire”: Sauce prepared from boiled cocoyam leaves

“Shitor”: Pepper sauce usually spiced and used as a complement to other dishes

### Coordination and case management

The case-patients were managed with intravenous fluids, Oral Rehydration Salts and Doxycycline at the district health center. Some of them were treated and discharged whilst others were referred to a nearby hospital for further management. All the patients who presented at the health facility were discharged within 48 hours. The District Health Management Team collaborated with the political leaders of the District and initiated a prompt investigation into the outbreak. However, food samples and clinical specimen from case-patients were not collected for laboratory investigation. The Eastern Regional Health Directorate was also notified in a timely manner. School authorities of two Senior High Schools in the vicinity assisted the team to interview and educate staff and students. The food vendor and her assistant were stopped from selling food pending the investigation report and decolonized after the laboratory investigation.

### Laboratory investigation

At both the health centre and the referral hospital, the health workers who had initial contact with the case-patients did not take specimens for laboratory investigation. Stool specimens obtained from the food vendor and her assistant were negative for intestinal parasites using microscopic examination of direct smear mount in saline and formol-ether concentration procedures. Stool cultured on thiosulfate-citrate-bile-sucrose agar (TCBS) plates at 37°C for 48 hours after enrichment in Alkaline Peptone Water was also negative for Vibrio cholerae. Dilutions of stool suspensions inoculated on mannitol salt agar at 37°C for 48 hours was also negative for *Staphylococcus aureus*. Nasopharyngeal specimens from the two food handlers showed gram-positive clustered cocci from β haemolytic golden yellow colonies on blood agar plate, which had been incubated for 24 hours at 35-37°C. The isolates tested positive for S.*aureus* using conventional biochemical tests such as catalase test and slide coagulase test. All food leftovers were disposed of before the team arrived on the field. The implicated food items were therefore not available for testing.

### Surveillance

Enhanced surveillance activities were ongoing prior to the outbreak as the district was responding to a cholera outbreak. Local health officials were on the high alert for any unusual events and community members were sensitized to report any unusual events to the health authorities. Both active and passive surveillance were used in gathering information during the outbreak. The food vendor, but not her assistant, had a valid food vendor's certification.

### Environmental assessment

The general environment was untidy particularly in the vicinity of the food joint. The ingredients used in food preparation were stored in a storeroom at the food vendors residence. There was no evidence of refrigeration of ingredients and leftover food items. There was no drainage system and wastewater from homes was flowing freely in the vicinity. A refuse disposal container was located about 100 meters from the food joint. There was no evidence of open defecation in the community.

## Discussion

Our results indicate a foodborne disease outbreak linked to food consumed from a popular community food joint. One of the implicated food items, “waakye” is a popular Ghanaian dish of boiled rice and beans and usually eaten with locally prepared pepper sauce called “shitor”. “Waakye” had been observed to be significantly associated with FBD in Ghana [14] though contaminated water source was the main item implicated in that outbreak. The features of this outbreak is consistent with toxin-producing pathogen contamination. Relatively longer incubation periods among those who consumed food earlier compared to those who ate later was indicative of infection caused by preformed toxins. A conclusive determination of the aetiologic agent of this outbreak was however challenging because of the missed opportunities in the management. The fact that local health officials did not collect leftover of food items from the food vendor as well as stool and vomitus samples from the case-patients for laboratory confirmation limited the extent to which a definite diagnosis could be made [9]. The linkage of enterotoxigenic pathogensto the outbreak was therefore based on compelling epidemiological evidence. In the absence of laboratory confirmation of the aetiologic agent in food samples and clinical specimens, a thorough evaluation of epidemiological and clinical characteristics of the outbreak becomes the useful alternative. The course of the outbreak demonstrates a point source without secondary cases. The only case of FBD observed on the 24^th^ of September 2014 could be unrelated to the outbreak especially as there was an ongoing cholera outbreak in the district at the time of this investigation. It is therefore possible that the case-patients had cholera or any other infection mimicking the FBD under investigation. It was however difficult to tell whether V. *cholera* was the cause of this outbreak since clinical specimens of case-patients were not tested. However, the symptoms and course of the illness exhibited by the case-patients were atypical of cholera infection. Also, the incubation period of 4 to 9.5 hours observed among case-patients in this study contrasts with 12 hours to 4.4 days incubation period of cholera proposed to guide epidemiological and clinical investigations [15]. With the exception of the index case who had some residual abdominal pain, all the other case-patients had complete resolution of the symptoms within 48 hours of onset, a feature not characteristic of cholera. Linking this outbreak to V. *cholera* would therefore be challenging considering the lack of correlation between the clinical features of cholera and those exhibited by the case-patients in this outbreak. Another finding worth evaluating was the nasopharyngeal carriage of *S. aureus* in the food handlers. Though both food handlers had *S. aureus* colonization, a feature commonly reported among food handlers [7, 16–18], diagnosis of staphylococcal foodborne disease was also not possible because of the missed opportunities of collecting clinical and food specimens for laboratory analysis. Conclusive diagnosis of staphylococcal foodborne disease is based on laboratory evidence of staphylococcal enterotoxins in food or isolation of *S.aureus* from food samples [7, 19]. However, these were not possible in this investigation as there were no food leftovers available for testing at the time of the outbreak investigation, a phenomenon known to hamper the determination of aetiologic agents in FBD outbreaks [9]. Clinical specimens were also not taken from the case-patients for laboratory testing. When the outbreak investigation team got to the field, all the case-patients had been discharged.

The general response to this outbreak was rigorous, however, initial steps by the local health authorities warrants capacity building for the health staff in response to outbreaks. The failure to obtain food leftovers for laboratory testing demonstrated a major gap in their response capacity. This shortcoming had been observed in a FBD outbreak in the Eastern Region [14]. The reasons for this could be lack of expertise and weak laboratory support in these resource-limited settings. The absence of a national FBD surveillance system spelling out specific tasks for health officials may be a contributory factor to these. Laboratory response in this outbreak was feeble probably because of the ongoing cholera outbreak in the district at the time of the investigation. The apparent similarities between the symptoms of this illness and that of cholera as well as the epidemiological link of the cases to the existing cholera outbreak in the district could explain the symptomatic management of the case-patients without further laboratory investigations. The self-limiting nature of the illness, the absence of secondary cases and the closure of the food joint pending the investigation report were critical in the control of the outbreak. The response to this outbreak should be interpreted within the context of the limitations posed by the missed opportunities created by the local health officials. Implicating “waakye” and “shitor” as the vehicle of transmission was not supported by laboratory analysis but based purely on epidemiological evidence. There was no leftover of the implicated food items for laboratory analysis as the outbreak investigation team arrived a few days after the outbreak when all the food leftovers had been disposed of. In addition, no clinical specimens were obtained from the case-patients to demonstrate evidence of pathogens. The opportunity for matching any offending pathogen isolated from the case-patients and food with isolates from the food handlers was therefore lost. The resource-limited setting of the outbreak also presented its own challenge of inaccessibility to advanced laboratory support. Since *S. aureus* is a member of the normal flora of the nasal cavity of humans [20, 21], which is its main ecological niche [22–25], its isolation without demonstration of enterotoxigenic strains was a limitation in implicating it as the cause of the FBD outbreak. Nonetheless, identification of the strains using enterotoxin kit reagent, Phage typing and Polymerase Chain Reaction techniques would at best be suggestive but inconclusive of staphylococcal foodborne disease since detection of staphylococcal enterotoxins from food or isolation of the organism from food leftovers and clinical specimens from case-patients was not possible. Isolation of the *S. aureus* from the food handlers only suggests but does not necessarily implicate it as the cause of the outbreak. A causative agent other than *S. aureus*, could be responsible for this outbreak, as the possibility of viral causes could also not be explored. These limitations have key implications on the response to this outbreak. Firstly, the definitive aetiologic agent of the outbreak is not known and therefore control of the outbreak was challenging. Secondly, specific interventions required to prevent a recurrence was lacking. Control and prevention activities were based presumptive treatment of case-patients and implementation of general standard hygienic practices. Other limitations include the fact that information on the complete cohort of patrons of the implicated food joint was unavailable as some had travelled out of the town and a few could not be traced because of poor address system. There was also a possibility of recall bias [26] where case-patients might have recalled differently from those who were not ill. Considering the fact there was an ongoing cholera outbreak during the period, differential misclassification [26] could not be ruled out. Residents who were ill from cholera could have been inadvertently classified as case-patients.

## Conclusion

A point source FBD outbreak occurred in Adeiso from 25^th^ to 26^th^ September 2014. It was a point source outbreak affecting community members mostly students between the ages of 20 to 39 years. The vehicle of transmission was probably locally prepared delicacies “waakye” and “shitor” most likely contaminated with toxin-producing organism. Prompt case management and rigorous outbreak investigation and response helped in controlling the outbreak. Health education on personal and environmental hygiene given to the community members and the food vendors was geared at preventing future outbreaks. Strengthening of the district's laboratories and capacity to response to unusual events has been recommended. When FBD is suspected, district health officials must promptly collaborate with the food vendors and take samples of food leftovers as well as clinical specimens from case-patients for laboratory investigation. An effective FBD surveillance system complemented by a strong laboratory capacity at all levels of the health sector is warranted to combat FBD.

### What is known about this topic

- Foodborne disease outbreaks are common but underreported.
- Conclusive diagnosis of FBD outbreaks are based on a combination of epidemiological, clinical and laboratory evidence.

### What this study adds

- This study highlights missed opportunities in FBD outbreak investigations and how these hinder response activities.
- The study illustrates the use of epidemiological evidence in investigating and responding to outbreaks in the absence of comprehensive laboratory techniques.
- It also highlights the need for instituting and implementing foodborne disease surveillance and capacity building of frontline health workers in resource poor settings.
